# Some seriously fishy research puts holes in movement barriers

**DOI:** 10.1093/conphys/coy017

**Published:** 2018-04-18

**Authors:** Sean Tomlinson

**Affiliations:** 1 School of Molecular and Life Sciences, Curtin University, Kent Street, Bentley, WA 6102, Australia; 2 Kings Park Science, Department of Biodiversity, Conservation and Attractions, Kattij Place, Kings Park, WA 6005, Australia

Human structures, like roads or spillways, can create barriers for animals that need to move between habitats and therefore can also impede conservation measures. And, as Essie Rodgers*et al*. (2017) highlight in their research, movement costs energy, and energetic costs can become rapidly limiting. However, ‘locomotor efficiency’ can be measured, and it turns out that locomotor efficiency of freshwater fishes can depend on whether they are swimming over a smooth vs. roughened surface. This is important because drainage culverts are typically built to help water pass under roads but are also typically built with smooth surfaces, which can prevent fish from passing through efficiently. Could this problem be solved if culverts, for example, are built with rough surfaces instead?

To figure this out, Rodgers and her team conducted a study to determine how well freshwater fishes could swim against currents of different speeds. They also compared upper critical swimming speeds when fish were swimming over smooth vs. roughened surfaces. It turned out that the fish could swim 26% faster when swimming over roughened surfaces. While this seems like such a simple finding, it has huge practical implications. Smooth culverts that are designed to maximize water runoff can become barriers, even if they are only 2m long. If the surfaces are rough, culverts can extend as long as 20 m without posing issues for traversing fishes.

The impressive value of this study is that it truly represents conservation physiology in action: there are quantifiable guidelines as to acceptable rates of water flow through drainage culverts, but engineering to meet these expectations is complex and expensive. With such a simple, yet elegant question, centred on a single physiological response, Rodgers and her team have provided ample evidence to guide conservation and wildlife management. In providing estimates on dispersal distances in the two fish species that they studied, Rodgers and her team were able to determine the length at which drainage culverts become barriers. They also suggested a very specific and affordable material with which to construct new culverts or modify existing culverts to reduce or overcome these barriers. This study on fish locomotion is a great example of recognizing a readily quantifiable, physiological basis to an ecological management problem, and the great evidence-based management policy that can arise from such investigations.

Engineering solutions to allow fauna to cross roads is a major research focus, and also a major cost in much of the developed world. In Australia, for example, millions of dollars can be added to construction costs to account for these issues. Empirical constraints are one of the first points to reduce costs and increase success in engineering solutions. A mechanistic understanding of the problem implies quantifiable management thresholds and testable metrics for success. The study by Rodgers and team has generated the evidence-base that will ultimately benefit the management challenge regarding dispersal barriers for freshwater fishes.

**Figure:**
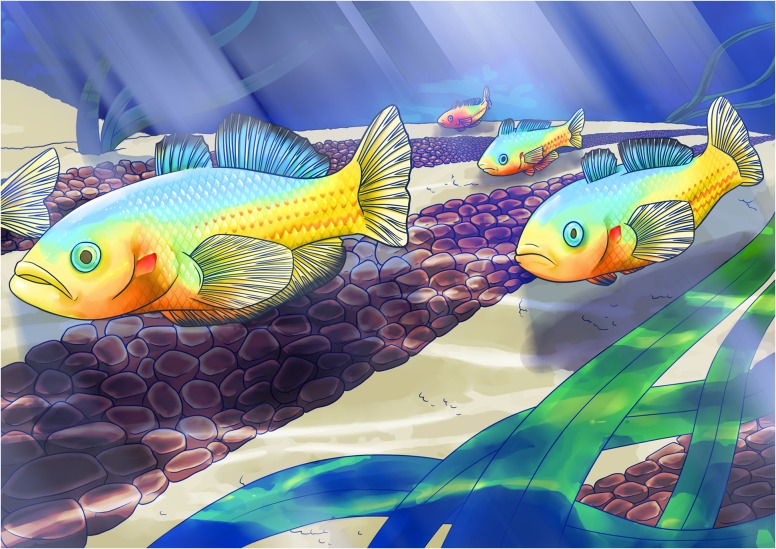


Illustration by Erin Walsh; Email: ewalsh.sci@gmail.com
